# Sodium-Dependent Vitamin C Transporter 2 (SVCT2) Expression and Activity in Brain Capillary Endothelial Cells after Transient Ischemia in Mice

**DOI:** 10.1371/journal.pone.0017139

**Published:** 2011-02-11

**Authors:** Burkhard Gess, Sevgi Sevimli, Jan-Kolja Strecker, Peter Young, Wolf-Rüdiger Schäbitz

**Affiliations:** 1 Department of Neurology, University Clinic Muenster, Muenster, Germany; 2 Neurologie Bethel, Evangelisches Klinikum Bethel, Bielefeld, Germany; Biological Research Center of the Hungarian Academy of Sciences, Hungary

## Abstract

Expression and transport activity of Sodium-dependent Vitamin C Transporter 2 (SVCT2) was shown in various tissues and organs. Vitamin C was shown to be cerebroprotective in several animal models of stroke. Data on expression, localization and transport activity of SVCT2 after cerebral ischemia, however, has been scarce so far. Thus, we studied the expression of SVCT2 after middle cerebral artery occlusion (MCAO) in mice by immunohistochemistry. We found an upregulation of SVCT2 after stroke. Co-stainings with Occludin, Von-Willebrand Factor and CD34 demonstrated localization of SVCT2 in brain capillary endothelial cells in the ischemic area after stroke. Time-course analyses of SVCT2 expression by immunohistochemistry and western blots showed upregulation in the subacute phase of 2–5 days. Radioactive uptake assays using ^14^C-labelled ascorbic acid showed a significant increase of ascorbic acid uptake into the brain after stroke. Taken together, these results provide evidence for the expression and transport activity of SVCT2 in brain capillary endothelial cells after transient ischemia in mice. These results may lead to the development of novel neuroprotective strategies in stroke therapy.

## Introduction

Ischemic stroke caused by cerebral artery occlusion results in the activation of a complex cascade of pathophysiologic events, including brain edema, blood-brain barrier disruption, oxidative stress and neuroinflammation [1]. These events crucially determine the extent of the final infarction [2]. Therapeutic interventions are remarkably limited and the number of patients afflicted with cerebral ischemia is steadily increasing. In spite of beneficial effects of thrombolysis, only a small percentage of acute stroke patients qualifies for this specific therapy [3]. Despite extensive experimental and clinical research, efforts to establish neuroprotective therapies in stroke patients have not been successful so far [4].

Ascorbic acid – also known as Vitamin C – has shown neuroprotective effects in neuronal cell cultures [5], [6], [7] and animal models of Alzheimer, Parkinson and Huntington disease [8], [9], [10]. In a mouse stroke model using middle cerebral artery occlusion (MCAO), Vitamin C has been shown to be cerebroprotective [11], [12]. Despite its promising role in neuroprotection, the transport pathway of Vitamin C into the brain is not entirely clear. Two studies have shown transport of Vitamin C across the blood-brain barrier in its oxidized form – dehydroascorbate – via Glucose Transporters (GLUT) [5], [13]. This has also been demonstrated for the blood-retinal barrier [14]. However, it is still debated whether this transport pathway is the physiological mechanism of Vitamin C transport into the brain [15]. Another possible pathway is transport of the reduced form of Vitamin C – ascorbic acid – via Sodium-dependent Vitamin C Transporters (SVCTs). In contrast to GLUTs, SVCTs are specific for Vitamin C and transport ascorbic acid by an active, sodium-dependent mechanism. Two types of SVCT have recently been cloned and characterized: SVCT1 and 2. SVCT1 is expressed mainly in tissues involved in whole-body homeostasis of Vitamin C like the kidney, liver and gastrointestinal tract. SVCT2 on the other hand is expressed predominantly in organs and tissues that functionally require ascorbic acid like the neuroendocrine organs, the lung, the peripheral and central nervous system [16], [17], [18]. In the brain, expression of SVCT2 has been shown in hypothalamic glial cells and neurogenic zones of the rat fetal brain [19], [20]. SVCT2 mRNA transcripts have been detected by fluorescence in situ hybridization after experimental cerebral ischemia in mice [21]. However, there were no studies so far on the expression of SVCT2 protein and transport activity of SVCT2 after stroke.

In this study, we analysed the expression, localization and activity of SVCT2 in mouse brain after middle cerebral artery occlusion (MCAO). We found expression of SVCT2 in brain capillary endothelial cells after MCAO in mice. Upregulation of SVCT2 occured in the subacute phase (2–5 days) after stroke. Furthermore, we show transport of radioactively-labelled ascorbic acid across the blood-brain barrier after MCAO in mice. Thus, we show for the first time expression and transport activity of SVCT2 in the blood-brain-barrier after transient ischemia in mice. These results could be of importance for the development of antioxidative therapies of ischemic stroke.

## Materials and Methods

### Ethics statement

All animal experiments were done in strict accordance with a protocol approved by the University of Muenster and the government of North-Rhine-Westphalia (Landesamt für Natur, Umwelt und Verbraucherschutz Nordrhein-Westfalen, AZ 87-51.04.2010.A273).

### Animal stroke model

Adult male mice (Jackson Laboratories) weighing 20–30 g were used in this study. Mice were allowed free access to water and food before surgery. Anesthesia was induced with 2% and maintained with 1% isoflurane in a mixture of 70% nitrous oxide and 30% oxygen. A modified standard intraluminal filament technique was used to induce transient focal cerebral ischemia [22], [23]. The left middle cerebral artery occlusion (MCAO) was induced by a 8-0 nylon monofilament (Ethilon; Ethicon, Norderstedt, Germany) and coated with silicon resin (Xantopren; Heraeus, Dormagen, Germany). Cerebral blood flow was continuously monitored using a laser Doppler probe (Periflux 5001; Perimed, Stockholm, Sweden) to verify ischemia and reperfusion. During the experiment rectal temperatures were maintained at 37°C±0,5°C with a thermostat-controlled heating pad. After 30 min, the filament was withdrawn to reperfuse the ischemic brain and the animals were allowed to recover from anesthesia. After the appropriate survival time, the animals were deeply anesthetized and perfused transcardially with 4% paraformaldehyde (PFA). Brains were rapidly removed from the skull, postfixed in 4% PFA (3 h), immersed in 10% sucrose overnight, embedded in TissueTek® (Sakura Finetek, Netherlands), frozen and stored at −80°C.

### Immunohistochemistry

Immunhistochemistry was performed in MCAO mice on 10 µm-thick frozen coronal sections using SVCT2 rabbit polyclonal antibody (1∶100, Santa Cruz) and an appropriate secondary biotinylated goat anti rabbit antibody (1∶100, Vectashield). Double fluorescent staining was performed using SVCT2 in combination with the Zonula occludens marker Occludin (1∶100, Zymed Laboratories), the neuronal marker NeuN (Chemicon), the glial marker GFAP (Sigma), the endothelial marker Von-Willebrand-Factor (1∶50, Santa Cruz) and CD34 (1∶100, BD Biosciences). Immunostaining was visualized with a fluorescent microscope (Leica DM microscop, Bensheim, Germany). Negative control sections were used without the primary antibody. For quantification, images were analysed with the software ImageJ (NIH). Orthogonal sections of confocal microscopy stacks were computed by the open source software BioImageXD (http://bioimagexd.net).

### Western blot

For western blot analysis, brains were dissected, the hemispheres separated and frozen immediately at −80°C. Hemispheres were incubated with lysis buffer (25 mM Tris, 1 mM NaVO3, 1% SDS and 2 mM EDTA) on ice for 45 min. and then homogenized with a Dounce homogenisator followed by multiple passes through a 21 gauge syringe. Lysates were then centrifuged at 4°C with 10,000 rgf for 15 min. Protein concentrations were determined by the Bradford method. Samples with 10 µg of protein were loaded onto 10% SDS-polyacrylamide gels. After electrophoresis, proteins were transferred to PVDF membranes (Millipore) in a wet blot chamber (Biorad) at 4°C over night. Membranes were stained with Ponceau Red (Biorad), de-stained with tris-buffered saline containing 0.05% Tween (TBST), blocked with 4% milk powder and incubated with primary antibodies SVCT2 (1∶1000) or β-Actin (1∶10000) at 4°C over night. Membranes were washed with TBST and incubated with HRP-conjugated secondary antibodies (1∶10000, Molecular Probes) at room temperature for one hour. Bound antibodies were visualized using chemiluminescence (Pierce) and x-ray films. Densitometric analysis of western blots was performed with the program ImageJ (NIH).

### Radioactive uptake assays

A solution of 2.5 µCi 14C-labelled L-ascorbic acid or dehydroascorbate (160 µg/ml) and 5 µCi 3H-labelled inulin (114 µg/ml) in 250 µl of 0,9% NaCl was injected into the tail vein by standard procedures. 30 minutes after injection, animals were sacrificed, brains quickly dissected, hemispheres separated and kept on ice. Hemispheres were lysed with 300 µl buffer containing 10 mM TrisHCl, 0.2% SDS and 2% Triton100. Brain tissue was then homogenised using a Dounce homogenisator followed by multiple passes through a 21-gauge syringe. Brain homogenates were added to 2 ml of β-scintillation liquid (Roth). β-radiation was then measured in a liquid scintillation counter (LKB Wallac).

### Data analysis

Data in text, tables, and figures are expressed as mean ± SD. Statistical comparisons among multiple groups were evaluated using two-way analysis of variance followed by Tukey post-hoc test for intergroup comparisons. For all statistical procedures, differences were considered significant at P<0.05.

## Results

### SVCT2 is expressed in brain capillaries in infarcted brain tissue after transient ischemia

**Figure 1:**
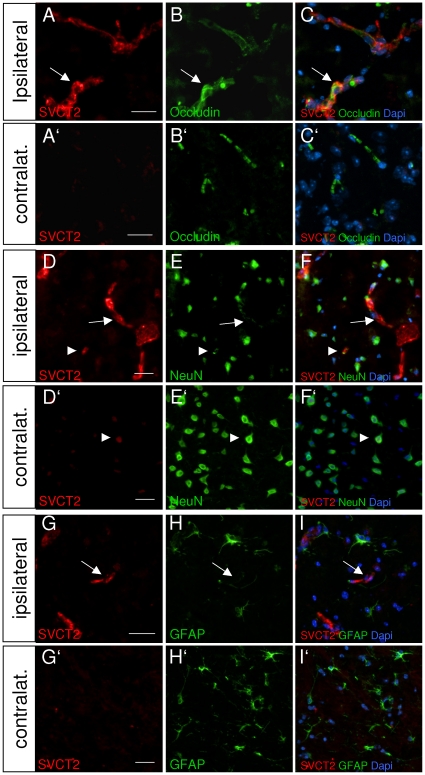
SVCT2 is upregulated after stroke and mainly localized in endothelial structures. Sections of brain tissue from mice with MCAO were stained with SVCT2 (red, left column) and co-stained with occludin (B, B'), NeuN (E, E'), GFAP (H, H'). Intense SVCT2- immunoreactivity was found in the infarct area (ipsilateral), compared to only weak expression on the contralateral side (compare A to A', D to D', G to G'). Co-immunohistochemistry with occludin showed colocalisation in linear, occasionally branched structures reminiscent of capillaries (A–C, D–F, G–I, arrows). Co-stainings with NeuN showed weak SVCT2-immunoreactivity in NeuN positive cells in both hemispheres (D–F, D'–F', arrowheads). Co-stainings with GFAP showed no SVCT2-immunoreactivity in GFAP-positive cells (G–I, G'–I'). Size bars: 10 µm.

To assess the expression of SVCT2 after middle cerebral artery occlusion, brain sections of mice were immunohistochemically stained with SVCT2 antibodies five days after MCAO. SVCT2 staining was visualized intensely in the infarct regions of ischemic mice, whereas the hemispheres contralateral to the lesion showed only weak immunoreactivity (Fig. 1, compare A to A', D to D', G to G'). SVCT2 staining in infarcted brain tissue appeared in short, linear, occasionally branched structures reminiscent of capillaries (Fig. 1, arrows). To analyse the cellular localization of SVCT2 immunoreactivity, co-stainings with the endothelial tight junction protein Occludin (Fig. 1A–C'), the neuronal marker NeuN (Fig. 1D–F') and the glial protein GFAP (Fig. 1G–I') were prepared. Co-localisation was found in co-stainings with Occludin in the infarcted brain tissue, but not in the contralateral, non-ischemic hemisphere (Fig. 1A–C'). SVCT2 was weakly expressed in some NeuN positive cells of both hemispheres (Fig. 1D–F', arrowheads) as expected from a previous study showing SVCT2 expression by neurons of healthy rats [24]. GFAP-positive cells showed no SVCT2 staining in either hemisphere (Fig. 1G–I'). Untreated control mice showed no SVCT2 staining brain endothelia in either hemisphere, consistent with previous reports [20], [24] (data not shown).

**Figure 2:**
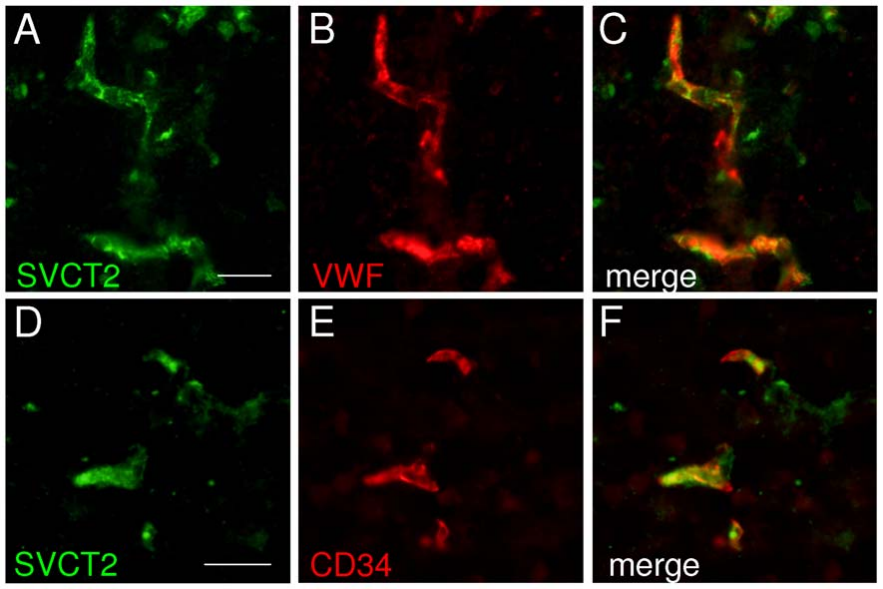
SVCT2 is localized in brain capillary endothelial cells after murine stroke. Brain sections of mice with focal cerebral ischemia were stained with SVCT2 and Von-Willebrand factor (vWF) antibodies (A–C) or SVCT2 and CD34 antibodies (D–F). SVCT2 colocalised with both vWF and CD34, indicating a localization in brain capillary endothelial cells. Size bars: 10 µm.

Occludin immunoreactivity is found in endothelial cells in tight junctions and in the cytoplasm. However, it is not specific for endothelial cells but is also found in epithelial tight junctions. Thus, we used antibodies against Von-Willebrand Factor (VWF) and CD34 to confirm endothelial localization of SVCT2 after stroke. Fluorescence microscopy showed co-localisation of SVCT2 and VWF (Fig. 2A–C) as well as SVCT2 and CD34 (Fig. 2D–F) in brain capillary endothelial cells in the infarcted area. In order to demonstrate co-localisation of SVCT2 and VWF specifically, double-immunolabeled sections were viewed with a confocal microscope. Confocal microscopy also showed co-localisation of SVCT2 and VWF in brain capillary endothelial cells in infarcted brain tissue (Fig. S1A–C). Three-dimensional analysis of confocal images confirmed colocalization in computed transverse sections (Fig. S1C).

Taken together, these data provide evidence for the expression of SVCT2 in brain capillary endothelial cells after MCAO in mice.

### Time-course of SVCT2 expression after MCAO

Pathological mechanisms after stroke occur in several phases characterized for example by excitotoxicity in the early phase, followed by inflammatory processes and apoptosis in later phases. We were interested in the time course of SVCT2 expression after MCAO to find out in which phase after stroke SVCT2 upregulation occurs.

**Figure 3:**
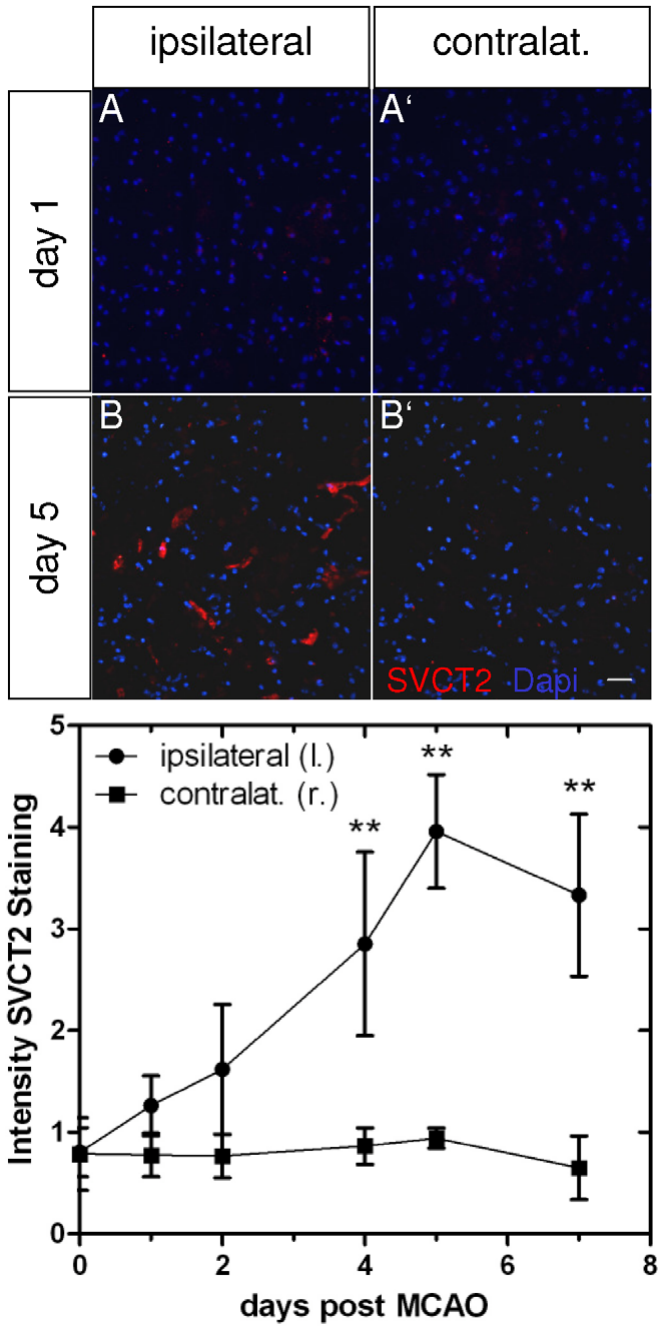
Time-course of SVCT2 immunohistochemistry after murine stroke. Brains of mice with a focal cerebral ischemia were dissected at day 0, 1, 2, 4, 5 and 7 after stroke and stained for SVCT2 (red) and nuclei (DAPI, blue). Ipsilateral (A–B) and contralateral (A'–B') hemispheres of day 1 and 5 are shown. An increase of SVCT2 immunoreactivity was found in the ipsilateral hemisphere at day 5 (B). Contralateral hemispheres (A'–B') showed only weak background staining. Quantification of immunohistochemistry showed a significant increase at day 4, a peak at day 5 and a decline at day 7 (C). Size bar: 10 µm. (** p<0.01, n = 4).

We studied SVCT2 expression by immunolabeling of sections from stroke mice at day 0, 2 and day 5 after MCAO. SVCT2 levels started to increase in the stroke area at day 2 and reached a peak at day 5 (Fig. 3B). On the contralateral side no increase in SVCT2 expression could be seen (Fig. 3B'). Quantification of immunohistochemistry showed a slight increase of SVCT2 immunoreactivity at day 1, further increasing at day 2, reaching significance at day 4, peaking at day 5 and declining again at day 7 (Fig. 3C).

**Figure 4:**
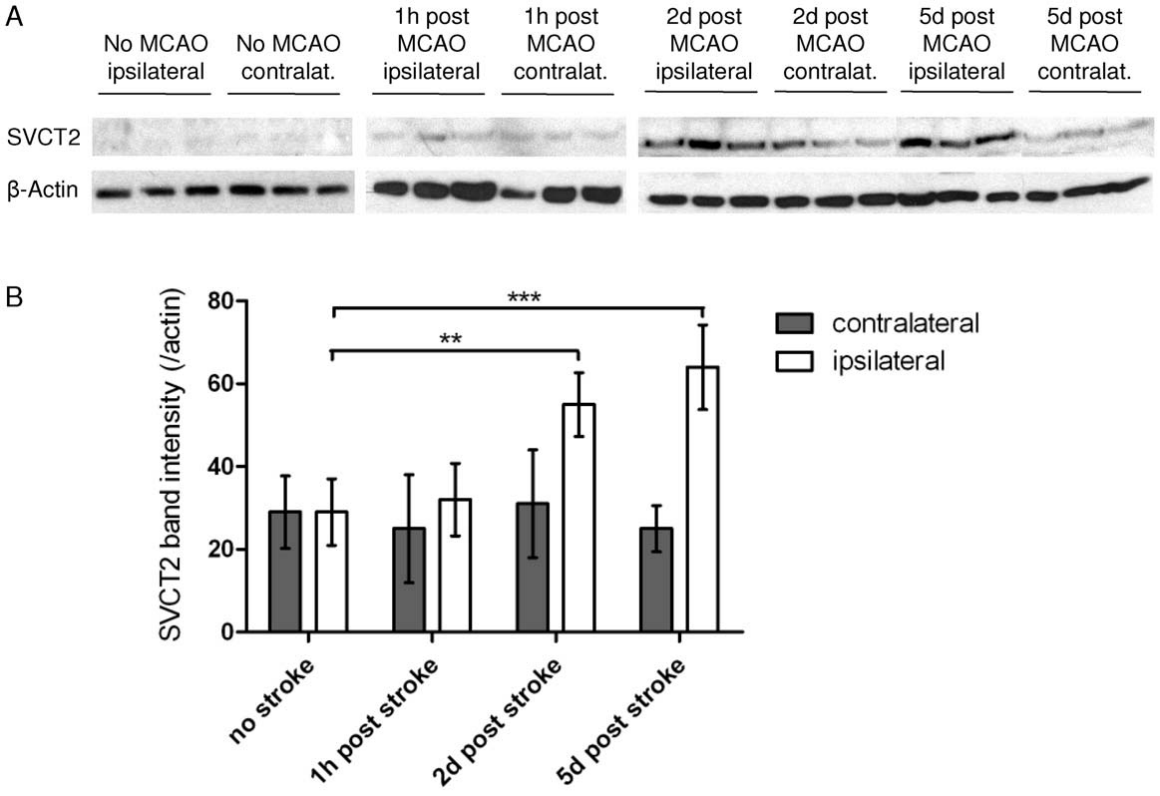
Time-course of SVCT2 protein levels after stroke. To assess the levels of SVCT2 on a whole-protein level, brains of stroke mice were dissected and lysed at day 0, day 2 and day 5 after stroke and analysed by western blot. Western blots showed only very weak SVCT2 bands in animals without stroke and on day 0 after stroke. An increase in SVCT2 bands can be seen in ipsilateral hemispheres 2 days after stroke and a further increase 5 days after stroke (A). Actin was used as a marker for protein loading (A). SVCT2 and actin bands were measured using image analysis software ImageJ (NIH). Intensities of SVCT2 bands were normalized by the corresponding actin band. Semi-quantitative analysis of western blot signals showed a significant increase of SVCT2 at days 2 and 5 in ipsilateral hemispheres (B). (** p<0.01, *** p<0.001, n = 3).

To assess the time-course of SVCT2 expression after MCAO on a whole protein level, we performed western blots of lysates from the ipsi- and contralateral hemispheres of operated animals at 0, 2 and 5 days after MCAO and untreated control animals. Western blot analysis showed that SVCT2 was upregulated in the infarcted hemisphere after stroke, whereas no increase could be observed in the contralateral hemisphere or in untreated control brains (Fig. 4A). Quantification of western blot signals, normalized by actin signals, showed that SVCT2 levels were not increased at day 0 (1 hour) after stroke; a slight increase could be observed at day 2 and a further increase at day 5 after stroke (Fig. 4B). Therefore, western blot analysis confirmed the results of the immunohistochemical studies.

These data show that SVCT2 expression is upregulated in the subacute phase at days 2–5 after murine stroke.

### Uptake of 14C-labelled ascorbic acid into brain tissue after MCAO

After analysing the localization and time-course of SVCT2 expression after transient ischemia, we were interested if SVCT2 was functionally active in the blood-brain-barrier after stroke as well. In order to test this, mice at day 0 and 5 after stroke as well as control mice without stroke were injected with radioactively- (14C-) labelled L-ascorbic acid. Since dehydroascorbate is known to be transported across the blood-brain-barrier via GLUT both in healthy mice and after stroke [25], [26], [27] - 14C-labelled dehydroascorbate was used as a positive control. 3H-labelled inulin was used as a negative control because inulin does not permeate the intact blood-brain-barrier.

**Figure 5:**
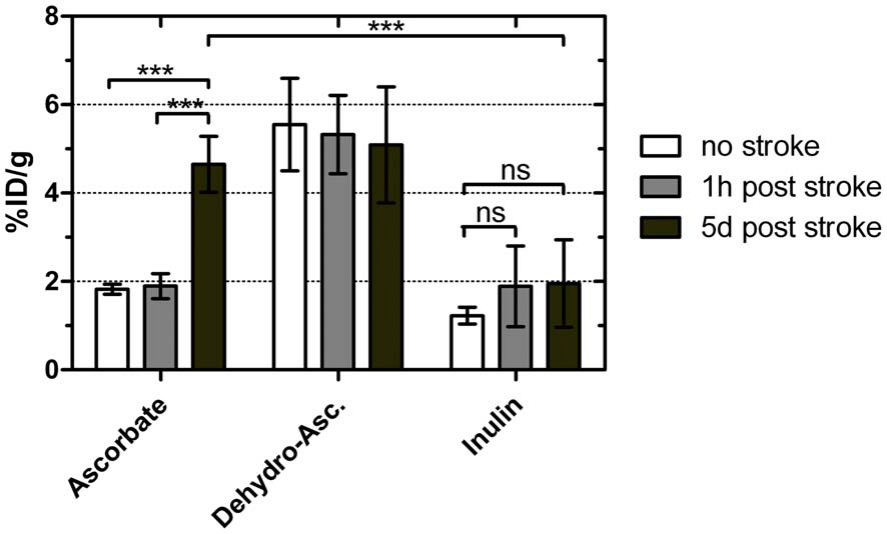
Uptake of 14C-labelled ascorbic acid into the brain is increased 5 days after stroke. Animals with focal cerebral ischemia and control animals without stroke were injected with 14C-labelled ascorbic acid or dehydroascorbate and 3H-labelled inulin. Radioactive uptake assays showed a significantly increased ascorbic acid uptake 5 days after stroke compared to animals without stroke or at day 0 after stroke. Dehydroascorbate was transported into the brain at similar rates before, at day 0 and day 5 after stroke. At day 5 ascorbic acid transport was not significantly lower than dehydroascorbate. Inulin uptake, as a marker for blood-brain-barrier integrity showed an insignificant trend towards an increase after stroke. (*** p<0.001, ns  =  non-significant, n = 4).

Analysis of radioactive uptake showed that there was no increase in ascorbic acid uptake into brain tissue of the infarcted hemisphere at the time point of day 0, but a significant increase at day 5 after stroke (Fig. 5). This finding is in line with the time-course of SVCT2 expression in our immunohistochemical and western blot analyses (see above). 14C-dehydroascorbate was readily transported into brain tissue at all time points (Fig. 5), as expected from previous reports [11], [13]. Dehydroascorbate is rapidly reduced to ascorbic acid intracellularly [28]. Thus, the radioactivity measured in brain after 14C-dehydroascorbate injection may actually come from 14C-dehydroascorbate that was already reduced intracellularly to 14C-ascorbic acid. Nonetheless, since reduction does not take place in the blood circulation, radioactivity after 14C-dehydroascorbate injection reflects Vitamin C transported into the brain as dehydroascorbate not as ascorbic acid.

The uptake-rates of 3H-inulin showed a non-significant trend towards an increase after stroke, indicating a possible slight perturbation of the blood-brain-barrier by the ischemic event. However, the differences in 3H-inulin uptake levels were insignificant and considerably smaller than those in 14C-ascorbic acid uptake. There was no difference in 3H-inulin uptake between day 0 and day 5 after stroke (Fig. 5). Therefore, the increased uptake of 14C-ascorbic acid 5 days after stroke cannot be explained by a destruction of the blood-brain-barrier.

Taken together, these data provide evidence for functional activity of SVCT2 transporters in vivo leading to active transport of ascorbic acid across the blood-brain barrier after stroke in mice.

## Discussion

In this study we provide evidence, for the first time, for an upregulation of SVCT2 protein expression after experimental stroke in mice. After stroke, SVCT2 was localized specifically in brain capillary endothelial cells immunoreactive for the tight junction marker Occludin and the endothelial cell markers VWF and CD34. The contralateral, non-ischemic hemisphere showed no immunoreactivity for SVCT2 in endothelial cells. Apart from endothelial cells, SVCT2 was weakly positive in neurons of both hemispheres, but not in astrocytes. The time-course of SVCT2 expression in immunohistochemistry and immunoblotting showed that SVCT2 was upregulated in the subacute phase at day 2–7, with a peak at day 5 after transient ischemia. Furthermore, we found a significant increase of L-ascorbic acid uptake into the brain at day 5 after MCAO, suggesting functional activity of SVCT2 in ascorbic acid transport across the blood-brain-barrier after stroke.

Our radioactive uptake studies showed transport of labelled dehydroascorbate as well as ascorbic acid into the brain after stroke. Dehydroascorbate was transported into the brain without stroke, in the acute phase and in the subacute phase after stroke, while ascorbic acid was significantly transported across the blood-brain-barrier only in the subacute phase after stroke. These data match the expression courses of the respective transporters – GLUT and SVCT2 – in brain capillary endothelia: GLUTs are constitutively expressed in the brain endothelium in healthy as well as ischemic brain tissue [25], [26], [27], whereas SVCT2, as we show in our time course analyses, is expressed in brain endothelium only in the subacute phase after stroke. As GLUTs are expressed in control conditions and after stroke, transport of dehydroascorbate into the brain could be found with and without stroke in our present study and previous studies by other investigators [11], [13]. It is unclear, however, if this dehydroascorbate transport plays a physiological role. Under physiological conditions, dehydroascorbate is found in blood only in small amounts compared to ascorbic acid [29], [30]. Since GLUTs are glucose transporters, dehydroascorbate competes with glucose for transport via GLUTs [31], [32]. Thus, at physiological glucose and dehydroascorbate concentrations, little transport of dehydroascorbate via GLUTs is to be expected [15]. Overall, the mechanism of Vitamin C uptake into the brain under physiological conditions is still under debate. It has been suggested that Vitamin C enters the brain through the choroid plexus and the cerebrospinal fluid [33]. Cultured choroid plexus epithelial cells show a sodium-dependent Vitamin C transport pathway and expression of SVCT2 [34]. Under physiological conditions, no expression of SVCT2 in brain capillary endothelial cells was detected in a study by Qiao et al. [35], though SVCT2-expression developped during the process of cell culturing – possibly attributable to oxidative stress. One study, on the other hand, showed transport of radioactively labelled ascorbic acid in isolated brain capillaries [36]. This study, however, provided no data on SVCT2 expression on the RNA or protein level. Our study presented here demonstrates expression of SVCT2 and transport of ascorbic acid in brain capillary endothelial cells only after stroke, not under physiological conditions. This supports the hypothesis that under physiological conditions ascorbic acid is transported via the choroid plexus but after stroke – and possibly in other pathological conditions – there may be ascorbic acid transport by SVCT2 across the blood-brain-barrier.

Since it is known that the blood-brain-barrier is disturbed after stroke, it has to be considered that a loss of blood-brain-barrier function may have caused the increase in ascorbic acid uptake after stroke. However, inulin, which cannot cross the intact blood-brain-barrier and, thus, is a marker of blood-brain-barrier breakdown, was only slightly, insignificantly increased after stroke. This indicates that the observed elevation of ascorbic acid uptake after stroke was not attributable to a reduction of blood-brain-barrier function.

Previous studies have demonstrated expression of SVCT2 in different cell types of the central and peripheral nervous system in vivo and in vitro. SVCT2 was shown to be expressed and functionally active in tanycytes - hypothalamic glial cells [20], brain stem stem cells, neuroblastoma cells, embryonic and adult neurons of different subtypes in rats and mice [7], [19], [24], [37]. The SVCT2 knockout mouse shows a severe phenotype with lung failure and brain hemorrhage leading to early postnatal death [38]. Brain ascorbic acid content in embryonal SVCT2-deficient mice was dramatically reduced [38]. In our previous studies, we found expression and activity of SVCT2 in Schwann cells and axons of peripheral nerves [39]. In one previous study, mRNA transcripts of SVCT2 were found in glial and neuronal cells in rats after stroke [21]. Our present study provides evidence for the first time for expression and transport activity of SVCT2 in the blood-brain barrier in vivo. SVCT2 shows a strict specificity for L-ascorbic acid, as D-ascorbic acid, dehydroascorbate and several other forms of Vitamin C are not transported [16]. In contrast to humans, mice are able so synthesize L-ascorbic acid in the liver. Whether Vitamin C is delivered nutritionally as in humans or by the liver as in mice, the expression and activity of Vitamin C transporters in functional end-organs may be the same. However, caution should be exercised in translation of results on Vitamin C metabolism from mice to humans.

The function of ascorbic acid in the central nervous system and specifically after stroke, however, remains elusive. Some studies have suggested a function of ascorbic acid in free radical scavenging and prevention of oxidative damage in brain tissue and endothelia [6], [7], [40], [41]. Interestingly, a study by Qiao et al. showed that SVCT2 was not expressed in brain capillary endothelial cells in untreated mice in vivo, but developed in cultured brain capillary endothelial cells, which the authors attributed to oxidative stress during the course of cell culturing [35]. After cerebral ischemia, oxidative stress is one of the factors leading to cell death by oxidation of various cell stuctures including membrane-lipids, proteins and DNA [42]. The vascular endothelium is considered one of the major targets of oxidative stress injury [42], [43], [44]. Thus, increased expression of SVCT2 in brain endothelia after stroke may have a role in the response to increased oxidative stress. This study used an ischemia-reperfusion model to induce a stroke in mice. Previous studies suggested that reperfusion was a major cause of oxidative stress after stroke [45]. Hence, reperfusion may be the actual trigger of SVCT2-upregulation in our study. On the other hand, there is one study on ischemia without reperfusion in cats, in which brain ascorbic acid fell markedly over the course of 24 hours after stroke [46]. This ascorbic acid decrease could also be a stimulus for SVCT2-upregulation after stroke. Since our model used ischemia-reperfusion it is not possible to differentiate between the effects of the initial ischemia and the subsequent reperfusion. Further studies will be necessary to clarify this aspect.

Oxidative stress parameters are significantly increased from 6 hours to 7 days after stroke [42], so it is a pathogenetic factor present at the time-point of 2–5 days after stroke, at which we found SVCT2 upregulation. Apart from oxidative stress, inflammatory processes are prominent at this time-point. Macrophages and neutrophiles show a maximum of brain infiltration 2–5 days, T-cells around 3–4 days after stroke [47], [48]. A function of ascorbic acid in protection of macrophages from oxidants generated by phagocytosis of cellular remains has been suggested [49]. Therefore, ascorbic acid uptake into the brain may be necessary for the function of inflammatory cells infiltrating the brain after stroke.

A previous treatment study showed a neuroprotective effect of dehydroascorbate but not ascorbic acid after stroke in mice [11]. In this study ascorbic acid and dehydroascorbate were administered right before stroke, 15 minutes and 3 hours after stroke, i.e. in the acute phase. Our data show that at these time points SVCT2 is not expressed in brain capillary endothelial cells and radioactively labelled ascorbic acid is not taken up into the brain yet. Thus, the time points of ascorbic acid treatment in the study by Huang et al. [11] may have been to early to show a beneficial effect of ascorbic acid on stroke in mice. Another study, however, did show a neuroprotective effect of ascorbic acid itself given right before stroke in monkeys [50]. Furthermore, dehydroascorbate is reduced to ascorbic acid intracellularly, possibly consuming antioxidants like NADPH and glutathione in cells. This may in turn have pro-oxidative effects. Hence, the question of dehydroascorbate versus ascorbic acid as a therapeutic approach to stroke is still controversial. Treatment studies administering ascorbic acid in the time window of SVCT2 expression from day 2 to 7 after stroke may be promising.

In summary, this study provides evidence for expression and function of SVCT2 in brain capillary endothelia after transient murine stroke. Ascorbic acid transported into the brain after stroke may have a role in oxidative stress protection or macrophage function. Further studies are necessary to assess the function of ascorbic acid after stroke and the mechanisms leading to SVCT2 upregulation. A treatment study administering ascorbic acid to mice with cerebral ischemia in the time window of SVCT2 expression after stroke is warranted on the basis of our data. Such studies may lead to the development of novel therapeutic strategies against stroke with the opportunity of a delayed time window for treatment initiation.

## Supporting Information

Figure S1 **Confirmation of endothelial localization of SVCT2 by confocal microscopy.** Sections stained with SVCT2 and VWF antibodies were viewed and photographed with a confocal microscope to confirm colocalization. SVCT2 staining (A), VWF staining (B), and the merged image (C) are shown. Computed orthogonal sections are shown to the right and bottom of the merged image (C). Colocalization of SVCT2 and VWF is shown in confocal images and computed orthogonal sections, confirming endothelial localization of SVCT2 after cerebral ischemia. Size bar: 25 µm.
(TIF)
