# Supplementary figures and images for: Sodium-Dependent Vitamin C Transporter 2 (SVCT2) Expression and Activity in Brain Capillary Endothelial Cells after Transient Ischemia in Mice
Source: PLoS One. 2011 Feb 11;6(2):e17139. doi: 10.1371/journal.pone.0017139 (PMC3037964; doi:10.1371/journal.pone.0017139)

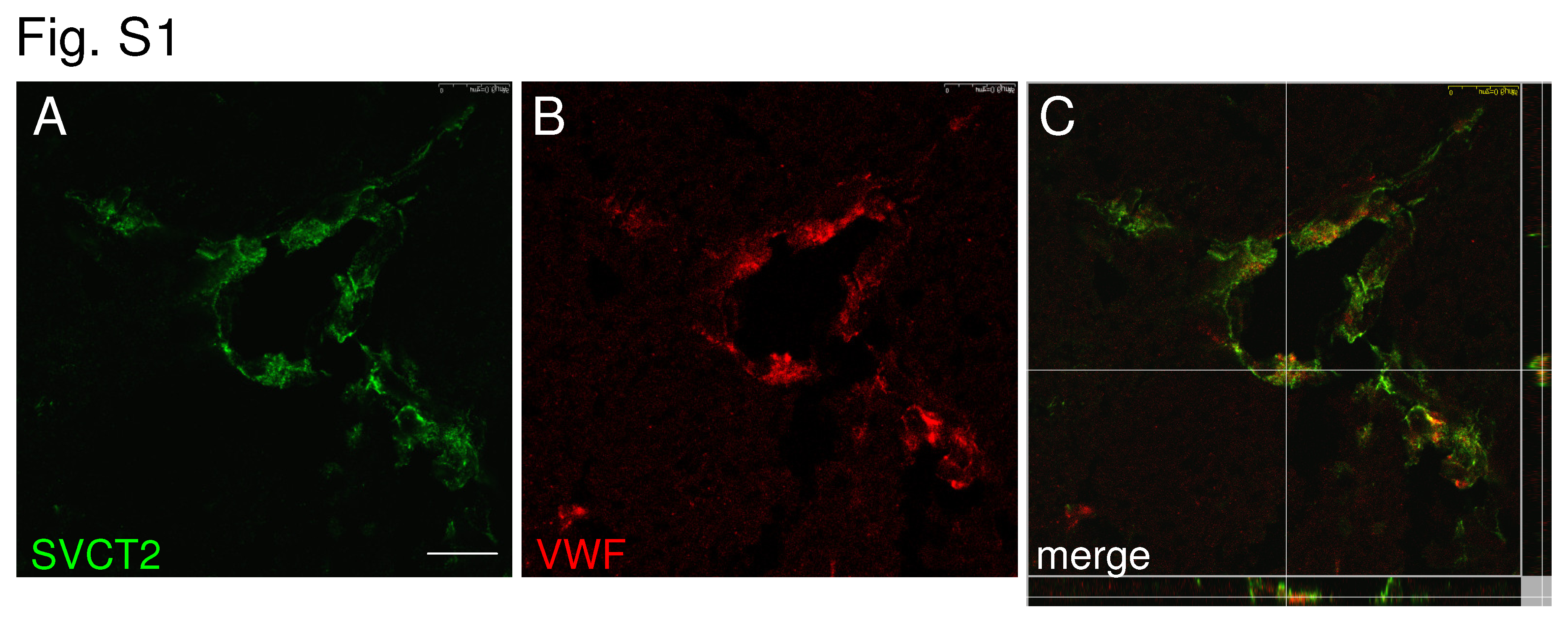

Supplement: Figure S1 — Confirmation of endothelial localization of SVCT2 by confocal microscopy. Sections stained with SVCT2 and VWF antibodies were viewed and photographed with a confocal microscope to confirm colocalization. SVCT2 staining (A), VWF staining (B), and the merged image (C) are shown. Computed orthogonal sections are shown to the right and bottom of the merged image (C). Colocalization of SVCT2 and VWF is shown in confocal images and computed orthogonal sections, confirming endothelial localization of SVCT2 after cerebral ischemia. Size bar: 25 µm. (TIF) [file pone.0017139.s001.tif]
